# Spectroscopic abnormalities in the pregenual anterior cingulate cortex in obsessive-compulsive disorder using proton magnetic resonance spectroscopy: a controlled study

**DOI:** 10.1186/s12888-023-05228-3

**Published:** 2023-10-10

**Authors:** Eliška Kosová, Dita Pajuelo, Iveta Fajnerová, David Greguš, Martin Brunovský, Pavla Stopková, Antonín Škoch, Petra Fürstová, Filip Španiel, Jiří Horáček

**Affiliations:** 1 National Institute of Mental Health, Klecany, Czech Republic; 2 Third Faculty of Medicine, Charles University, Prague, Czech Republic; 3 MR Unit, Department of Diagnostic and Interventional Radiology, Institute for Clinical and Experimental Medicine, Prague, Czech Republic

**Keywords:** Obsessive-compulsive disorder, N-acetyl aspartate, Choline-containing compounds, Creatine, Myo-inositol, Magnetic resonance spectroscopy

## Abstract

**Background:**

The main aim of the present study is to determine the role of metabolites observed using proton magnetic resonance spectroscopy (1H-MRS) in obsessive-compulsive disorder (OCD). As the literature describing biochemical changes in OCD yields conflicting results, we focused on accurate metabolite quantification of total N-acetyl aspartate (tNAA), total creatine (tCr), total choline-containing compounds (tCh), and myo-inositol (mI) in the anterior cingulate cortex (ACC) to capture the small metabolic changes between OCD patients and controls and between OCD patients with and without medication.

**Methods:**

In total 46 patients with OCD and 46 healthy controls (HC) matched for age and sex were included in the study. The severity of symptoms in the OCD was evaluated on the day of magnetic resonance imaging (MRI) using the Yale-Brown Obsessive-Compulsive Scale (YBOCS). Subjects underwent 1H-MRS from the pregenual ACC (pgACC) region to calculate concentrations of tNAA, tCr, tCho, and mI. Twenty-eight OCD and 28 HC subjects were included in the statistical analysis. We compared differences between groups for all selected metabolites and in OCD patients we analyzed the relationship between metabolite levels and symptom severity, medication status, age, and the duration of illness.

**Results:**

Significant decreases in tCr (U = 253.00, p = 0.022) and mI (U = 197.00, p = 0.001) in the pgACC were observed in the OCD group. No statistically significant differences were found in tNAA and tCho levels; however, tCho revealed a trend towards lower concentrations in OCD patients (U = 278.00, p = 0.062). Metabolic concentrations showed no significant correlations with the age and duration of illness. The correlation statistics found a significant negative correlation between tCr levels and YBOCS compulsions subscale (cor = -0.380, p = 0.046). tCho and YBOCS compulsions subscale showed a trend towards a negative correlation (cor = -0.351, p = 0.067). Analysis of subgroups with or without medication showed no differences.

**Conclusions:**

Patients with OCD present metabolic disruption in the pgACC. The decrease in tCr shows an important relationship with OCD symptomatology. tCr as a marker of cerebral bioenergetics may also be considered as a biomarker of the severity of compulsions. The study failed to prove that metabolic changes correlate with the medication status or the duration of illness. It seems that a disruption in the balance between these metabolites and their transmission may play a role in the pathophysiology of OCD.

## Background

Obsessive-compulsive disorder (OCD) is characterized by the presence of obsessions, compulsions, or most commonly both. Obsessions are unwanted thoughts or images (e.g., images of harming someone, thoughts of being contaminated, and thoughts of behaving in a way that violates one’s morals). Compulsions are repetitive behavior or mental acts that a person feels the need to do in response to an obsession (e.g., checking their behavior, washing hands, repetitive praying, thinking good thoughts to undo or replace bad thoughts) [1]. OCD has a lifetime prevalence of 2–3% in the general population without any sex differences in the adult population [2]. The severity of symptoms in OCD is measured with a specific scale called the Yale-Brown Obsessive-Compulsive Scale (YBOCS). The first-line treatment for OCD is cognitive-behavioral therapy (CBT) and serotonin re-uptake inhibitors (SRI). CBT is the most effective and evidence-based form of psychotherapy for OCD. As a rule, a higher dosage of SRIs is used for treatment of OCD rather than for other anxiety disorders, or major depression. Approximately 50% of patients with OCD fail to fully respond for the first-line treatment. Evidence-based pharmacological augmentation includes the use of antipsychotics, clomipramine, and glutamatergic agents [3]. For the treatment resistant forms of OCD, neuromodulation procedures are possible such as transcranial direct-current stimulation (tDCS), repetitive transcranial magnetic stimulation (rTMS), deep brain stimulation, and ablative procedures [4].

Pathophysiological mechanisms of OCD are connected with abnormalities in the cortico-striatal-thalamo-cortical circuit (CSTC) and have been replicated in previous neuroimaging studies [5, 6]. Specifically, hyperactivity during neutral or resting state in the orbitofrontal cortex (OFC), anterior cingulate cortex (ACC), and striatum, which is highlighted during symptom provocation and attenuated after successful treatment [7]. Although some data implicate other brain regions in the pathophysiology of OCD, the CSTC circuit remains the core in the psychopathology of OCD. The ACC plays a role in error detection and the monitoring and processing of conflicting information, yet its involvement in the pathophysiology of OCD has also been suggested [8, 9]. According to the results of Allman et al., the pregenual ACC (pgACC) contains a high density of the Von Economo Neurons (VENs) [10]. VENs are large bipolar neurons located in two specific areas in the central nervous system (CNS), i.e., the fronto-insular cortex and the limbic anterior area, in humans and great apes. Their morphology suggests involvement in rapid action in the CNS in comparison to pyramids cells, which send more detailed and complex information. VENs are implicated in several neuropsychiatric disorders (early stages of fronto-temporal dementia, agenesis of corpus callosum, schizophrenia etc.). VENs and their dysfunction are connected with diminished empathy, social awareness, and problems with self-control [10].

**Fig. 1:**
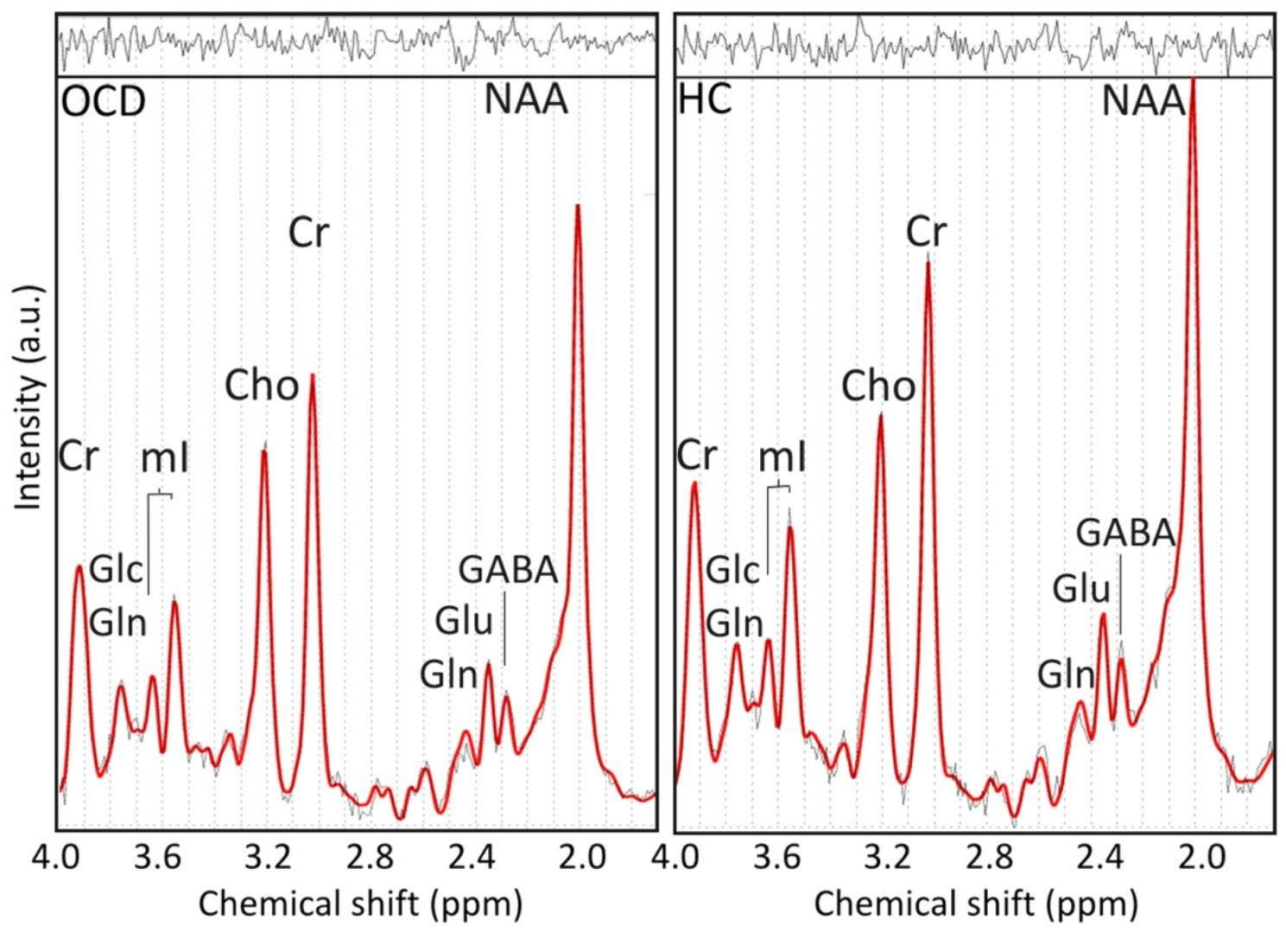
An example of a proton 1H-MRS spectrum measured using single voxel spectroscopy with short echo time from the anterior cingulate cortex in an obsessive-compulsive disorder patient (no.1917) and a healthy control (no. CHB1528) *Legend*: a.u.: arbitrary unit; ppm: parts per million; OCD: patient with obsessive-compulsive disorder; HC: healthy control; Cr: total creatine; GABA: gamma-aminobutyric acid; Glc: glucose; Gln: glutamine; Glu: glutamate; mI: myo-inositol; Cho: choline-containing compounds; NAA: total N-acetylaspartate.

Proton magnetic resonance spectroscopy (1H-MRS) allows in vivo measurement and quantification of metabolite concentrations such as N-acetyl aspartate, creatine/phosphocreatine, choline-containing compounds, and myo-inositol. To-date, it is the only method that allows non-invasively molecular structures to be studied in vivo [11]. N-acetylaspartate (NAA) and N-acetylaspartylglutamate (NAAG) are considered as markers of neuronal viability and density. NAAG is a molecule with a neurotransmitter like function on certain glutamate receptors, which is synthesized from NAA and glutamate [12]. Due to their overlapping peaks, NAA and NAAG are evaluated together as total NAA (tNAA) [7, 13]. The tNAA signal is the highest signal in the water-suppressed proton MR spectra (for illustration see Fig. 1), which makes this metabolite one of the most reliable markers in 1H-MRS measurement [14]. Creatine and phosphocreatine together are called total creatine (tCr). tCr is considered as a marker of cerebral bioenergetics [7, 12, 13]. The main contribution to the signal of choline-containing compounds is produced by phosphocholine, glycerophosphocholine, and choline, (total Cho; tCho). tCho reflects cell membrane constituents and abnormal membrane turnover [7, 12, 13]. Myoinositol (mI) is considered as a glial cell marker, which is connected to the osmoregulation of astrocytes. Increased mI probably reflects a glial activation and proliferation (known as the index of “glial metabolism”). In addition, mI is suggested to be a major osmolyte in the CNS, important for the integrity of the cells [12, 13]. However, its exact function remains unclear [7].

Previous studies of metabolite changes in the pgACC in OCD patients show inconclusive results. Some studies show lower tNAA levels in the ACC or medial frontal cortex (mFC) in OCD patients compared to healthy controls (HC) [15–17]. Gnanavel et al. compared tNAA levels in the ACC between OCD patients, a family-controlled group, and HC. The study showed that tNAA levels were significantly lower in the OCD group compared to the family control group, which also had lower tNAA levels in comparison to HC. Tükel et al. confirmed significantly lower tNAA/tCr in the ACC in the OCD group than HC [18]. Zheng et al. found significantly lower tNAA levels in the pgACC in a group of OCD patients with comorbid skin-picking disorder (SPD) in comparison to HC [19]. In the aforementioned study, the levels of tNAA were not different in patients with OCD with or without SPD. Niels de Joode et al. found no significant difference in the levels of tNAA in the dorsal ACC, but his team confirmed that patients with childhood onset OCD had lower tNAA levels than patients with adult onset OCD [20]. Several studies, however, showed no relevant differences in levels of tNAA in the ACC in OCD patients compared to HC [12, 21–24]. Zhu et al. and Batistuzzo et al. also found no significant differences of tNAA/Cr, tCho/Cr, and mI/Cr between OCD and HC groups in the medial pre-frontal cortex (mPFC) [25, 26]. Most of the studies also showed no differences in tCho, tCr, mI, and their ratios in the ACC between OCD and HC groups [12, 15, 18, 21–24, 26–28]. Compared to these studies, however, Gnanavel et al. showed higher tCho and mI levels in the ACC in OCD patients compared to a family control group, which had higher levels in comparison to HC [16]. Yücel et al. also showed a trend towards higher mI concentrations in the right ACC (rostral and dorsal part; p = 0.054) in OCD patients compared to HC [27]. A study involving children and adolescents (11–18 years old) proved lower mI levels in the ACC in patients [29]. However, Lázaro et al. found no relevant differences in ACC in an OCD group of children and adolescents (9–17 years old) compared to HC [30].

Previous studies also analyzed the relationship between metabolite levels and the severity of symptoms. Ebert et al. found a negative correlation between tNAA in the ACC and severity of the illness measured by the YBOCS [15]. Tükel at al. found a negative correlation between the YBOCS total score and tNAA/tCr, tCho/tCr, and mI/tCr ratios in the ACC [18]. O’Neill found a significant negative correlation between levels of tCr and tCho, and the severity of symptoms on the YBOCS and Montgomery–Åsberg Depression Rating Scale (MADRS) [22]. Bédard et al. found no relevant relationships between metabolite levels and severity of symptoms on the YBOCS, but confirmed a significant negative relationship between levels of anxiety on the Beck Anxiety Inventory (BAI) and tNAA/tCr in the ACC [21]. Other studies found no relevant correlation between the severity of symptoms in OCD patients and levels of tNAA, tCr, tCho, and mI in the pgACC (or in the ACC) [12, 16, 23, 27]. Metabolite concentrations also did not correlate with either duration of the illness or age of the enrolled OCD patients [12, 15, 16, 23].

No significant differences in metabolite levels in the ACC between patients with or without medication were found [27, 29]. Salles Andrade et al. found a positive correlation between tNAA/Cr and SRIs scores, and a negative correlation between tNAA/Cr and antipsychotic scores [28], but these findings did not survive statistical correction.

Although a number of studies regarding biochemical changes in the ACC have been published, the pathophysiology of OCD is still unknown. The studies conducted to-date yield conflicting results, often because they differ in the chosen inclusion criteria, applied methodology, or the selected region of CNS. We believe that the inconsistent results in the literature often arise also from the inclusion of low-quality MRS data without correcting for water content and calculating only concentration ratios. Taking these facts into account, we focused in our study on careful patient selection, placement of the 1H-MRS volume of interest (VOI) in the ACC, data quality control, and calculation of metabolite concentrations with corrections for water content in the VOI, which is essential for detecting low metabolic changes in OCD patients. The strict inclusion criteria of quality of MR spectra only allowed for tNAA, tCho, tCr, and mI evaluation. The glutamatergic metabolites were not evaluated because of the small sample size. A comparison of metabolite concentrations in the ACC between OCD patients and HC as well as between subgroups of patients with and without medication will contribute to a better understanding of metabolism in the ACC, related OCD pathophysiology, and changes associated with treatment. With regard to the previous literature, we expect lower levels of tNAA in the pgACC in OCD subjects vs. HC. We also expect increased levels of tCho and tCr in the pgACC with regard to accelerated metabolism in the pgACC in OCD patients. Furthermore, we hypothesized that deflections in metabolism would correlate with the severity of symptoms measured by the YBOCS and duration of illness.

## Methods

### Participants

In total, 46 patients (in-patients and out-patients) diagnosed with OCD according to ICD-10 [31] and DSM-IV [32] criteria, and 46 HC matched for age and sex were included in the study and underwent an MR examination. Exclusion criteria for all of the subjects were a concurrent severe medical disease, organic mental disorder, mental retardation, severe head injury, undergone neurosurgery procedure, substance abuse, lifetime history of psychosis, mood disorders, and any other severe mental disorder. HC must have no history of any mental disorder or psychotropic medication use. Patients with OCD were either without medication or with a stable dosage of antidepressant medication for at least 4 weeks.

### Ethical statement

The study was approved by the local ethical committee and informed consent was obtained from all of the enrolled subjects.

### Clinical assessments

The severity of symptoms in the OCD patients was evaluated on the day of magnetic resonance imaging (MRI) using the YBOCS [33]. Patients were recruited at the National Institute of Mental Health during an ongoing therapeutical program as inpatients or in daily stationary care. All the enrolled patients were symptomatic on the day of MRI. A total of 13 patients from the OCD group were on stable medication at least 4 weeks before the study with SSRI antidepressants (Sertraline n = 7; Citalopram n = 1; Escitalopram n = 2; Paroxetine n = 1; Fluvoxamine = 1), once augmented with Trazodone medication on a stable dosage. In addition, 13 were drug-free or the medication was discontinued at least 5 days before the measurement, in the case of two patients the medication status was not available. Only short-acting Zolpidem was allowed 1 day before the measurement for insomnia. On the day of MRI, no medication affecting the CNS was allowed.

### MR imaging

All subjects underwent an MR examination on a 3T Magnetom Prisma scanner (Siemens Medical Systems, Erlangen, Germany) equipped with a 64-channel volume head coil. The MRI protocol included T1-weighted sagittal images obtained using a three-dimensional (3D) magnetization-prepared rapid gradient-echo (MPRAGE) sequence (echo time (TE)/repetition time (TR)/Inversion time (TI)/number of acquisitions (NA) = 2.34 ms/2400ms/1000ms/1, iPAT = 2, resolution 1 × 1 × 1 mm). MR images were visually assessed by neuroradiologists to preclude the presence of lesions. One patient was excluded from the study due to abnormal MR findings. The MR images were further used for 1H-MRS localization purposes.

### Proton MR spectroscopy

**Fig. 2:**
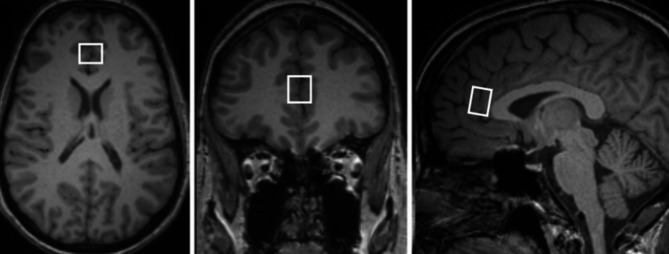
The positions of spectroscopic voxel in the pregenual anterior cingulate cortex (pgACC) on T1-weighted images of a patient with OCD.

The 1H-MRS protocol included single voxel spectroscopy (SVS) measurements from the pregenual anterior cingulate cortex. Spectra were obtained using the PRESS sequence: TE/TR/NA = 30ms/5000ms/64 with (or 1 without) water suppression, nominal voxel volume 3.8 ml. The MR images were used for the localization of VOIs. The VOI (20 × 12 × 16 mm^3^) in the pgACC was placed midsagittally, anterior to the genu of the corpus callosum. The VOI was positioned exactly on the rostral margin of the corpus callosum as the perpendicular axis to the anterior commissure–posterior commissure (AC–PC) line (Fig. 2). Spectra were evaluated using LCModel software [34]. A water signal was used as an internal calibration for the calculation of the metabolic concentration. Concentrations of tNAA, tCr, tCho, and mI were evaluated. Metabolic values were corrected for water content in each VOI ([35], equation No.8). This procedure requires information about the proportion of gray matter, white matter, and cerebrospinal fluid in each VOI ([35], equations No. 5–7), which was obtained by MPRAGE image segmentation using an SPM8 program [http://www.fil.ion.ucl.ac.uk/spm/software/spm8/] and an in-house Java-based tool for obtaining average tissue values from segmentation maps in the region of the examined VOI. Corrections for relaxation times were not made because their effect is small (< 4%) when using short TE and sufficiently long TR [35] and it was not necessary for the purposes of the study.

**Fig. 3:**
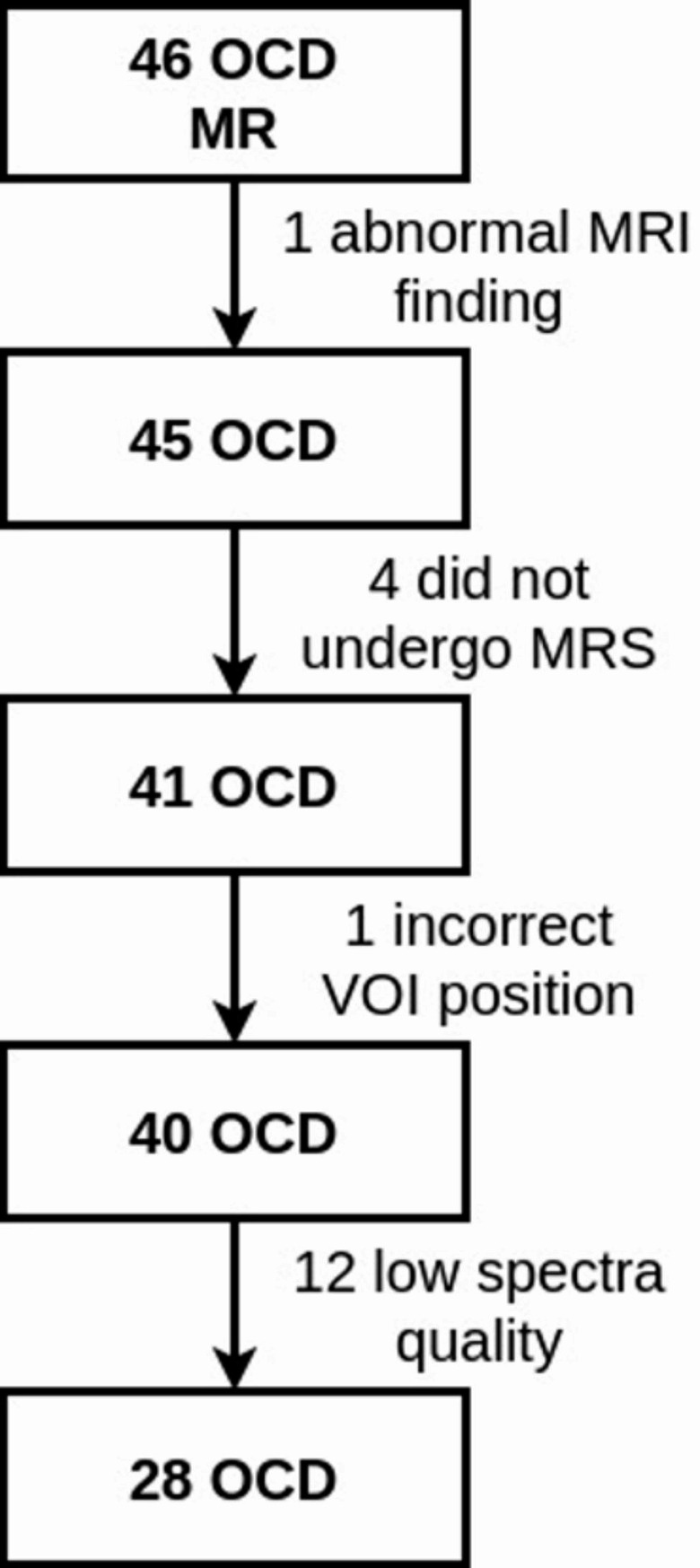
The exclusion graph in OCD group

Only 41 patients underwent 1H-MRS as four were unable to endure the entire examination protocol. The data from one of the patients were excluded because of incorrect VOI positioning. Only spectra that passed visual quality control with a signal-to-noise ratio above 10 and half-width at half maximum (FWHM) of the water signal below 0.08 ppm were included in the statistical analysis (28 OCD and 28 HC subjects, see Fig. 3) to ensure reliable metabolite quantification. Twelve patients were excluded because of FWHM > 0.08 (inadequate shim: 4, overlapping or duplicated signals because of patient movements: 2) or spectral artifact (4) or inadequate water suppression (2). The spectra from all 46 HC passed the spectral quality control. From this original HC group, twenty-eight pairwise sex/age matched healthy controls were selected according to the number of patients included in the statistical analysis.

In all remaining spectra used for statistical analysis the Cramer-Rao Lower Bound (CRLB) of the spectral fit did not exceed 9%.

### Statistical analysis

For demographic characteristics, descriptive statistical methods were used. A Chi-squared test was used for analysis of gender distribution in each group. Continuous variables were reported as medians with interquartile ranges (IQR). The Mann-Whitney U test was used for particular intergroup differences. The Mann-Whitney U test was also used to determine intergroup differences in the means of current (with/without) and the dominant dimensional type of clinical symptoms, where the most prevalent were “washing/fear of contamination” (n = 15) and “checking/harm” (n = 11). The groups with only one subject, i.e., “ordering/symmetry” and “mixed symptomatology” were not included in the final analysis. The correlation between metabolite levels and clinical characteristics (YBOCS, YBOCS obsession subscale, YBOCS compulsion subscale) was assessed by Spearman’s rank correlation coefficient. Spearman’s rank correlation was also used for determination of any relationship between age and duration of illness and levels of selected metabolites. The statistical analyses were performed using the Statistica program, version 13.0.

## Results

### Sample characteristics

Table 1 presents the general demographic and clinical characteristics of the study participants included in the statistical analysis. The OCD patient and healthy control groups did not differ in means of age or sex distribution. The patients showed a mean YBOCS score of 21.71 (± 7.41), mean obsession subscale of 10.96 (± 3.74), and compulsions subscale of 10.75 (± 4.21). The patients’ symptoms were evaluated with the YBOCS as subclinical symptoms (n = 1), mild (n = 4), moderate (n = 13), severe (n = 8), or extreme (n = 2) OCD symptomatology. Evaluation of dominant dimension types of clinical symptoms revealed prevalent “washing/fear of contamination” (n = 15) and “checking/harm” (n = 11). One patient presented “ordering/symmetry” (n = 1) and one patient mixed symptomatology. Subgroups of patients with (6 M/7 F, mean age 31.0 ± 4.4) and without (5 M/8 F, mean age 34.5 ± 10.0) medication did not differ in the means of age (Mann-Whitney U = 78; p = 0.75) or sex distribution (Chi-square χ2 = 0.16, p = 0.691).

### Characteristics of the tissue composition in VOIs

The distribution of GM, WM and CSF in the VOI in the OCD and HC groups did not statistically differ (Table 2).

**Table 1:** Demographic and clinical characteristics in the analyzed group of healthy controls and OCD patients and their medication status

Variable	OCD group (n = 28)			HC group (n = 28)			Group comparison OCD vs. HC	
	Mean	SD	Min-Max	Mean	SD	Min-Max	t-test/ χ^2^ test	p
**Age (years)**	32.6	7.8	22.1–52.6	32.9	7.9	22.6–52.8	-0.166	0.869
**Sex**	15 females/ 13 males			15 females/ 13 males			0.000	1.000
**Psychotropic medication**	with 13/ without 13/ 2 NA 2			with 0/ without 28				
**Duration of OCD (years)**	11.5	9.5	0.3–42.0					
**YBOCS**	21.71	7.41	5.00–39.00					
**Obsessions**	10.96	3.74	5.00–20.00					
**Compulsions**	10.75	4.21	0.00–20.00					

*Legend*: OCD: obsessive-compulsive disorder; HC: healthy controls; SD: standard deviation; NA: not available; YBOCS: Yale-Brown Obsessive-Compulsive scale; χ^2^ test: chi-squared test.

**Table 2:** Mean distribution of GM, WM and CSF in the VOI and their standard deviations

%	GM	WM	CSF
OCD group	72 ± 4	7 ± 5	21 ± 4
HC group	70 ± 6	9 ± 7	21 ± 4

### Metabolic differences between OCD and HC groups

**Fig. 4:**
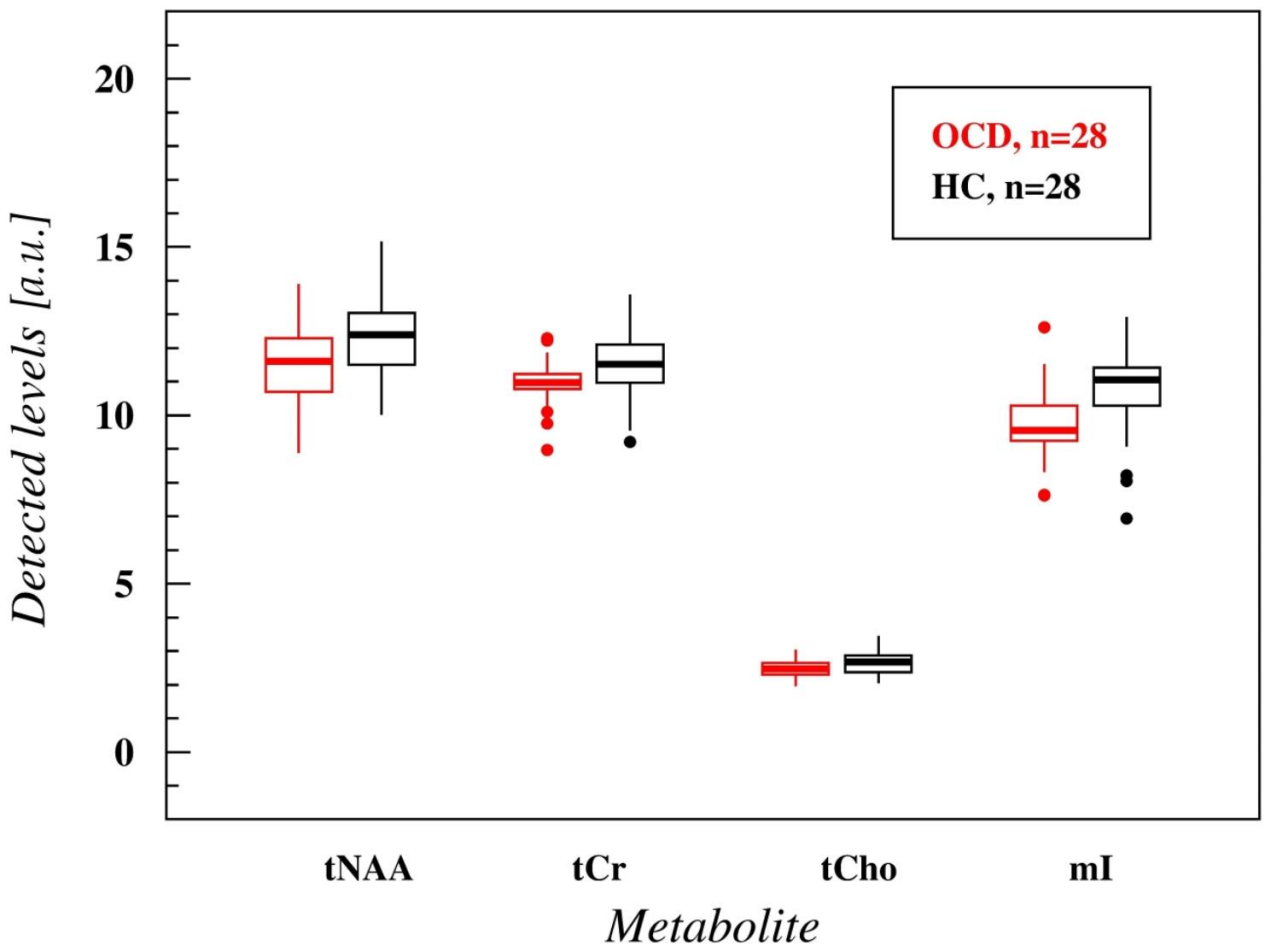
tNAA, tCr, tCho, and mI concentrations in the obsessive-compulsive disorder patients and healthy controls in the pregenual anterior cingulate cortex *Legend*: OCD: obsessive-compulsive disorder; HC: healthy control; HC: healthy control; tNAA: total N-acetyl-aspartate; tCr: total creatinine; tCho: total choline; mI: myo-inositole.

A significant decrease in tCr (U = 253.00, p = 0.022), and mI (U = 197.00, p = 0.001) was observed in the pgACC in the OCD compared to the HC group. No statistically significant differences were observed in the case of tNAA and tCho; however, tCho revealed a trend towards lower concentrations in the OCD patients compared to HC (U = 278.00, p = 0.062) (see Table 3; Fig. 4).

### Metabolic findings in the OCD subgroup

Metabolic concentrations did not correlate significantly with the age of subjects or duration of illness. The correlation statistics found a significant negative correlation between tCr levels and the YBOCS compulsions subscale (cor = -0.380, p = 0.046). tCho levels showed a trend to a negative correlation (cor = -0.351, p = 0.067) with the YBOCS compulsions subscale (see Table 4). Subgroups with and without medication did not reveal any significant metabolic differences (see Table 5). Our study demonstrates no significant differences in metabolite concentrations between dimensional OCD symptoms (“washing/fear of contamination” n = 15; “checking/harm” n = 11; tNAA: p = 0.259, U = 60.00; tCho: p = 0.148, U = 54.00; tCr: p = 0.574, U = 71.00; mI: p = 0.148, U = 54.00).

**Table 3:** Metabolite levels in the pregenual anterior cingulate cortex in the patients with OCD and healthy controls

Metabolites	OCD (n = 28)		HC (n = 28)		U value	p value
	Mean (SD) [mM]	Median [mM]	Mean (SD) [mM]	Median (SD) [mM]		
**tNAA**	11.69 (1.22)	11.61	12.37 (1.30)	12.40	281.00	0.69
**tCr**	11.02 (0.75)	10.97	11.53 (1.00)	11.53	253.00	0.022*
**tCho**	2.47 (0.28)	2.47	2.66 (0.38)	2.68	278.00	0.062
**mI**	9.72 (1.10)	9.55	10.67 (1.37)	11.06	197.00	0.001*

*Legend*: OCD: obsessive-compulsive disorder; HC: healthy controls; SD: standard deviation; tNAA: total N-acetyl-aspartate; tCr: total creatine; tCho: total choline; mI: myo-inositole; U value: value of U-statistic; *significant between-group difference p < 0.05.

**Table 4:** Metabolite levels in the pregenual anterior cingulate cortex in the OCD subgroup in dependency of the YBOCS, YBOCS obsessions subscale, and YBOCS compulsions subscale

Metabolites	YBOCS		YBOCS-O		YBOCS-C	
	p-value	cor	p-value	cor	p-value	cor
**tNAA**	0.665	0.086	0.321	0.195	0.871	-0.032
**tCr**	0.125	-0.297	0.496	-0.134	0.046*	-0.380
**tCho**	0.304	-0.201	0.840	0.040	0.067	-0.351
**mI**	0.620	-0.098	0.853	0.037	0.478	-0.140

*Legend*: OCD: obsessive-compulsive disorder; tNAA: total N-acetyl-aspartate; tCr: total creatine; tCho: total choline; mI: myo-inositol; YBOCS: Yale-Brown Obsessive-Compulsive Scale; YBOCS-O: Yale-Brown Obsessive-Compulsive Scale, obsessions subscale; YBOCS-C: Yale-Brown Obsessive-Compulsive Scale, compulsions subscale; cor: correlation coefficient; *significant between-group difference p < 0.05.

**Table 5:** ACC metabolite levels in the OCD subgroups in the pregenual anterior cingulate cortex with or without medication

1H-MRS metabolites	Without med. (n = 13)		With med. (n = 13)		U value	p value
	Mean (SD) [mM]	Median [mM]	Mean (SD) [mM]	Median [mM]		
**tNAA**	11.76 (1.30)	11.39	11.54 (1.26)	11.61	83.00	0.960
**tCr**	10.82 (0.94)	10.79	11.10 (0.45)	10.98	69.00	0.448
**tCho**	2.42 (0.29)	2.47	2.50 (0.28)	2.47	75.00	0.650
**mI**	9.66 (1.40)	9.27	9.80 (0.87)	9.77	71.00	0.511

*Legend*: ACC: anterior cingulate cortex; OCD: obsessive-compulsive disorder; HC: healthy control; SD: standard deviation; tNAA: total N-acetyl-aspartate; tCr: total creatine; tCho: total choline; mI: myo-inositole; U value: value of U-statistic; *significant between-group difference p < 0.05.

## Discussion

The available literature shows a convincing correlation between OCD pathology and neuroimaging findings in CSTC. These changes in pre-selected areas are usually described as hypermetabolism, or hyperactivity of neurons. Pathological activity in these regions is thought to be intuitively connected with higher metabolic needs for located neurons, and also neighboring glial cells and the extracellular matrix. Despite these general assumptions, mostly based on PET/SPECT results [5, 6], metabolic results from 1H-MRS studies, including our study, do not support the hypermetabolism hypothesis [12, 15, 18, 19, 21–24, 26].

The main finding of our study is a significant decrease in the levels of tCr and mI and a trend towards decreased tCho in the OCD group in comparison to HC. This finding does not correspond with our expectations based on published papers that OCD patients reveal accelerated metabolism in the ACC manifested by increased tCho and tCr [16, 27]. Gnanavel et al. confirmed increased tCho and mI levels in the ACC in OCD patients compared to both HC and a family control group [16], and Yücel et al. proved a trend towards higher concentrations of mI in the right ACC compared to HC [27]. However, metabolite concentrations in these studies were not corrected for water content in the examined VOI [36], which reduces the result reliability. Incorporating this correction when quantifying metabolite concentrations is essential for the detection of small metabolic changes expected in OCD, yet very few studies addressing OCD take this into account.

On the other hand, our findings correspond with the previously published study involving children and adolescents, which proved lower levels of mI [29]. This study adjusted metabolic concentrations to water content in the ACC as our study did. Our study showed decreased tCr and mI. tCr is considered as a marker of cerebral bioenergetics and its lower levels in the OCD group may support the paradigm of abnormal metabolism, which is described in the whole CSTC and in the pgACC as well. In addition, mI is a metabolite connected with glial metabolism, osmotic function in CNS, and plays the role of an intracellular second messenger. Its abnormal concentration is usually connected with altered cellular homeostasis. Abnormality in mI metabolism is also well documented in other psychiatric disorders [37]. In the 1990s, there were several placebo-controlled clinical trials evaluating the effectiveness of orally administered inositol in a variety of neuropsychiatric disorders with promising results. Fux et al. showed clinical improvement on the YBOCS in OCD (cross-over design, oral administration of inositol 18 g/day vs. placebo), but the results did not reach statistical significance [38]. Another clinical trial from the same team failed to prove its clinical efficacy as an add-on treatment to SSRI [39] and was never administered to common clinical practice. Our study also proved no differences in concentrations of tCho, which is in line with the majority of previous studies [12, 19, 22, 23]. However, we found a trend towards decreased tCho in the pgACC (U = 278.00, p = 0.062) in our OCD group. tCho is considered a marker of membrane synthesis or degradation [11]. Deflections in tCho levels in the ACC may support the hypothesis of abnormal membrane turnover in this region. Moreover, taking into account all the metabolic changes, we could hypothesize that OCD patients may suffer from abnormal glial cell metabolism. This hypothesis would be interesting for future research.

Surprisingly in our study, we found no significant differences in the levels of tNAA in the pgACC between the OCD and HC groups. This finding corresponds with several previous studies [12, 19, 22–24], but fails to prove our hypothesis of lower levels of tNAA in the pgACC. The role of NAA in the CNS is complex and its concentrations correspond with neuronal viability. NAA is decreased in diseases that involve neuronal loss, damage, and destruction [15], which seems to not be the case of OCD. Previous important findings concerning NAA are regional reduction in NAA concentration and NAA/Cr in clinical dementia, in epilepsy in the affected hemisphere, in the multiple sclerosis, and in mass lesions in CNS [14]. Normal NAA concentrations in the pgACC in our OCD group may be explained by active exposure therapy status (the ongoing therapeutical program), which may change the local neurochemistry in the pgACC [20, 23], or that the neuronal cells may be intact by OCD.

Another important finding that must be emphasized is a significant negative correlation between tCr levels and the YBOCS compulsions subscale, also found by O’Neill et al. [22]. Together with the finding of lower tCr levels in the OCD group, this implicates an important relationship of tCr levels with OCD symptomatology. As mentioned above, tCr is a marker of cerebral bioenergetics and may be a biomarker of the severity of compulsions in OCD patients. Also, the relation between tCho levels and the YBOCS compulsions subscale shows a trend towards a negative correlation, and highlights the hypothesis of abnormal membrane turnover in the pgACC connected with the severity of compulsions. A significant negative correlation between tCho and YBOCS was also confirmed by O’Neill et al. [22].

Our study showed no significant correlations between metabolite levels and age and duration of illness. These findings correspond to those of previous studies [12, 15, 16, 23, 29]. Studies on the differences in metabolite concentrations in relation to medication status are rare. Two studies are in line with our result as they showed no relevant differences between groups with or without medication [27, 29]. A study by Salles Andrade et al. confirmed some relevant findings (see the introduction), but they did not survive statistical correction [28]. Our study also demonstrates no significant differences in metabolite concentrations between dimensional OCD symptoms (“washing/fear of contamination” n = 15; “checking/harm” n = 11). Literature on this theme is generally missing.

The main problems of almost all the previous studies are the different methodologies applied and differences in sample sizes (recruiting of approximately 10–40 patients). In our study, we enrolled 46 OCD and 46 HC subjects. However, only 28 OCD patients and 28 HC passed the final inclusion criteria. Post-hoc, we analyzed the sample sizes for determination of a 10% change for each selected metabolite with 80% power. For tNAA, 15 subjects in each group are needed at a minimum, for tCho 20, for tCr 7 subjects, and for mI 18 subjects in each group. We enrolled sufficient sample sizes for the determination of promising and relevant differences in each metabolite. Based on this post-hoc analysis, we may consider our main outcomes as being fundamental.

The advantage of our study is the measurement on a 3T MR scanner, which allows unambiguous separation and detection of selected neurometabolites in a specific region. Only those spectra with a signal-to-noise ratio above 10 and FWHM of the water signal below 0.08 ppm were included in the analysis. However, no subject was excluded solely on the basis of SNR < 10. The spectra with SNR < 10 also had FWHM > 0.08 ppm. Selected spectral quality criteria were derived from articles by Kreis [39] and Jiru et al. [40]. We believe that the inconsistent results in the literature may arise from the inclusion of low-quality MRS data, among other things. The relatively higher number of patients excluded from the study based on poorer quality spectra may be striking, but their spectra had FWHM > 0.08 ppm resulting in overlapping metabolic signals and therefore unreliable metabolic value calculations. The individual reasons for excluding these patients are given in the Methods section. The dimensions of the VOI (20 × 12 × 16 mm^3^) were chosen according to the size of the ACC to reduce potential contamination from surrounding WM, which in our case reached on average only 7% of voxel volume in the OCD and 9% in the HC group (see Table 2). The volume of VOI is also large enough to ensure a sufficient SNR (28 ± 8 in OCD and 31 ± 8 in the HC group). The metabolic concentrations were also corrected for water content in the examined VOI, a key procedure for obtaining reliable metabolic concentrations and for the detection of small metabolic changes expected in OCD.

The strength of our study also lies in the strict inclusion criteria regarding the patients’ medication. Enrolled patients were either on stable medication (n = 13) at least 4 weeks before the study with SSRI medication, or without medication (n = 13). The medication was discontinued in all subjects at least 5 days before the MRI measurement. This period was chosen taking into account the half-life of the drugs used (Sertraline T_1/2_ = 23–26 h, Citalopram T_1/2_ = 33 h, Escitalopram T_1/2_ =27–32 h, Paroxetine T_1/2_ = 21–24 h, Fluvoxamine T_1/2_ = 15–16 h, and Trazodone T_1/2 =_ 5–13 h). Nearly five times the serum half-life is sufficient to minimize their peripheral effect and also their fluctuation in the CNS. There was also no other facultative medication used prior to the measurement, only short-acting Zolpidem was allowed on the day before for insomnia.

The presented study has several limitations. Mainly, the acquired data were only from the pgACC, not from the other brain regions that are also important in the neurobiology of OCD. Secondly, a methodological weakness is the combined medication status. For future studies, it will be more accurate to divide the OCD group into drug-naïve and drug-exposed patients. It is quite intuitive that the exposure of the patients to medication, but also to therapeutical methods (ongoing therapeutical program), may bring changes in metabolite levels in the examined area. Thirdly, glutamatergic metabolites were not evaluated because of the insufficient sample size. The present study aimed especially to provide accurate quantification (which is its great strength) for precise detection of small changes in the tNAA, tCr, tCho and mI and therefore relatively strict criteria of spectra quality (FWHM < 0.08 ppm and SNR > 10) were applied. Glutamatergic metabolites are multiplets with overlapping signals and the CRLB of the spectral fit are high and therefore even more strict criteria should be assessed to ensure reliable quantification. In the case of Glx, the FWHM should be decreased to < 0.05, which would exclude even more subjects, resulting in a very small sample size. Although relative CRLB in % are now widely used for quality filtering, their use is not so straightforward as it can produce potential bias in data sets [41]. Therefore, we used what in our view are more objective criteria for assessing spectral quality (FWHM). Fourthly, a correction for multiple comparison was not accounted for in the correlation statistics. Therefore, these results should be interpreted cautiously. Furthermore, although 1H-MRS is able to measure in vivo metabolite concentrations in the examined region, it is not able to provide detailed information about neurochemical processes in the CNS [7]. Despite these limitations, we consider MR spectroscopy to still be the most suitable non-invasive method for addressing the aim of the presented study.

Although we have chosen strict inclusion criteria, it would be advisable to adjust them further in future studies while maintaining a sufficiently large sample of subjects in the study groups. Furthermore, it would be interesting to measure other areas associated with the pathophysiology of OCD. Combined imaging methods such as diffusion-weighted imaging, diffusion-tensor imaging, and MR perfusions, may provide a more detailed view of the neurochemistry in OCD and may lead to a better understanding of this complex disorder, which usually brings a challenge in daily clinical practice. These studies may also open up the field of scientific interest in this mostly overlooked psychiatric disorder.

## Conclusion

This study confirmed abnormal metabolism in the pgACC in OCD patients. A significant decrease in tCr and mI concentrations in the pgACC in the OCD group compared to HC was found. Moreover, tCr levels showed a significant negative correlation with the severity of compulsions subscale in the YBOCS in the OCD group. These findings implicate an important relationship between tCr levels and OCD symptomatology. tCr is a marker of cerebral bioenergetics and may be a biomarker of severity of compulsions in OCD patients. No statistically significant differences were found in tNAA and tCho levels; however, tCho revealed a trend towards lower concentrations in OCD patients compared to HC. tCho levels also showed a trend towards negative correlation with the YBOCS compulsions subscale. This metabolic profile may indicate changes in glial cell metabolism in the pgACC rather than neuronal damage or dysfunction. As a correction for multiple comparison were not accounted for in the correlation statistics, these results should be interpreted cautiously.

Understanding the complex functions of metabolites such as total N-acetylaspartate, creatine, choline-containing compounds, and myo-inositol, which may be detected in vivo by 1H-MRS techniques, is surely more complicated, and needs to be understood not as a static function. But still, it is a good start for future analysis to understand molecular processes in OCD. Better diagnostics in the future and targeted treatment options may provide more detailed information about this challenging diagnosis. Additional research with more precisely chosen sample groups and improved methodology is still required to validate these observations.

## Data Availability

The datasets used and/or analyzed during the current study are available from the corresponding author on reasonable request.

## Abbreviations

ACC: Anterior cingulate cortex
AC–PC: Anterior commissure–posterior commissure
BAI: Beck Anxiety Inventory
CBT: Cognitive-behavioral therapy
CNS: Central nervous system
CSTC: Cortico-striatal-thalamo-cortical circuit
FWHM: Half-width of the water signal
HC: Healthy controls
IQR: Interquartile ranges
MADRS: Montgomery–Åsberg Depression Rating Scale
mFC: Medial frontal cortex
mI: Myo-inositol
mPFC: Medial pre-frontal cortex
MPRAGE: Three-dimensional magnetization-prepared rapid gradient-echo
MRI: Magnetic resonance imaging
MRS: Proton magnetic resonance spectroscopy
NA: Number of acquisitions
NAA: N-acetylaspartate
NAAG: N-acetylaspartylglutamate
OCD: Obsessive-compulsive disorder
OFC: Orbitofrontal cortex
pgACC: Pregenual ACC
rTMS: Repetitive transcranial magnetic stimulation
SPD: Skin-picking disorder
SRI: Serotonin re-uptake inhibitors
SVS: Single voxel spectroscopy
tCho: Total choline-containing compounds
tCr: Total creatine
tDCS: Transcranial direct-current stimulation
TE: Echo time
TI: Inversion time
tNAA: Total N-acetyl aspartate
TR: Repetition time
VENs: Von Economo Neurons
VOI: Volume of interest
YBOCS: Yale-Brown Obsessive-Compulsive Scale
